# Genomic Insights into Triple-Negative and HER2-Positive Breast Cancers Using Isogenic Model Systems

**DOI:** 10.1371/journal.pone.0074993

**Published:** 2013-09-23

**Authors:** Prakriti Mudvari, Kazufumi Ohshiro, Vasudha Nair, Anelia Horvath, Rakesh Kumar

**Affiliations:** 1 McCormick Genomic and Proteomics Center, School of Medicine and Health Sciences, the George Washington University, Washington, District of Columbia, United States of America; 2 Department of Biochemistry and Molecular Medicine, School of Medicine and Health Sciences, the George Washington University, Washington, District of Columbia, United States of America; Thomas Jefferson University, United States of America

## Abstract

**Introduction:**

In general, genomic signatures of breast cancer subtypes have little or no overlap owing to the heterogeneous genetic backgrounds of study samples. Thus, obtaining a reliable signature in the context of isogenic nature of the cells has been challenging and the precise contribution of isogenic triple negative breast cancer (TNBC) versus non-TNBC remains poorly defined.

**Methods:**

We established isogenic stable cell lines representing TNBC and Human Epidermal Growth Factor Receptor 2 positive (HER2+) breast cancers by introducing HER2 in TNBC cell lines MDA-MB-231 and MDA-MB-468. We examined protein level expression and functionality of the transfected receptor by treatment with an antagonist of HER2. Using microarray profiling, we obtained a comprehensive gene list of differentially expressed between TNBC and HER2+ clones. We identified and validated underlying isogenic components using qPCR and also compared results with expression data from patients with similar breast cancer subtypes.

**Results:**

We identified 544 and 1087 statistically significant differentially expressed genes between isogenic TNBC and HER2+ samples in MDA-MB-231 and MDA-MB-468 backgrounds respectively and a shared signature of 49 genes. By comparing results from MDA-MB-231 and MDA-MB-468 backgrounds with two patient microarray datasets, we identified 17 and 22 common genes with same expression trend respectively. Additionally, we identified 56 and 78 genes from MDA-MB-231 and MDA-MB-468 comparisons respectively present in our published RNA-seq data.

**Conclusions:**

Using our unique model system, we have identified an isogenic gene expression signature between TNBC and HER2+ breast cancer. A portion of our results was also verified in patient data samples, indicating an existence of isogenic element associated with HER2 status between genetically heterogeneous breast cancer samples. These findings may potentially contribute to the development of molecular platform that would be valuable for diagnostic and therapeutic decision for TNBC and in distinguishing it from HER2+ subtype.

## Introduction

Breast cancer is the most commonly diagnosed cancer among women worldwide [1]. In the United States, one out of every three cases of cancer diagnosed in women is that of the breast and associated malignancy is the second largest causes of cancer deaths [2]. Although breast cancer is claimed to have a higher prevalence among women from the developed part of the world, this statistics is rapidly changing. The incidence of the disease is on the rise even in developing countries, where the cumulative risk for women below 75 years of age and mortality rate is almost equivalent to the rate found in the developed countries [1]. The phenotypic and clinical manifestations of the disease vary widely among women, and various cancer subtypes show wide range of responses to different treatment modalities. The stage, grade and status of three therapeutically relevant receptors, estrogen receptor alpha (ER), progesterone receptor (PR) and human epidermal growth factor receptor 2 (HER2) are the main determinants of tumor response to most of the current treatments, and therefore, are the major factors in planning optimal therapy [3].

In the past decade, we have witnessed an active investigation of heterogeneity of breast cancer at the molecular level through various high throughput approaches. Derived from a large collection of tumors, these studies have classified breast cancer into five major subtypes based on expression pattern of an ‘intrinsic gene set’ signature [4,5]. These subtypes include luminal A and B, basal-like, HER2 overexpressing and normal breast like and are named according to the markers expressed by the corresponding cell types. These molecular classes not only differ in the expression levels of ER, PR and HER2 but also in disease prognosis [6]. The luminal subtypes show higher expression of ER and have a favorable prognosis and basal-like tumors have absence or low levels of the three receptors and in general, exhibit poor prognosis. These studies point to the likelihood that different breast cancer subclasses might stem from different cellular types based on origin. Over the years, a number of studies have validated and refined such signatures [7,8,9,10,11,12,13,14]. It is generally believed that different breast tumor subtypes represent distinct disease entities and may require personalized treatment modalities for an effective outcome. Despite multitude of studies on breast cancer expression signatures and their proclaimed robustness, the biological relevance of these signatures remains to be firmly established and this is an area for further improvement.

The status of ER, PR and HER2 is routinely assessed prior to deciding treatment options in general for breast cancer. Two of the most common treatment regimens include anti-estrogens like Tamoxifen or Fulvestrant or aromatase inhibitors for ER+ tumors and monoclonal antibody Herceptin (Trastuzumab) for HER2+ tumors. However, for TNBC that lacks both ER and HER2, there is no targeted therapy so far and the only option is non-specific and highly toxic chemotherapy or radiation therapy [15]. Unlike other subtypes of breast cancer, TNBC commonly affects younger (< 50 years) pre-menopausal women. It is a very aggressive form of breast cancer with majority of the deaths occurring within the first five years of diagnosis [16]. The relapse rates and the prognoses for these patients are very poor even after treatment [17]. Therefore there is a pressing need and growing research interest to understand how TNBC, which comprises approximately 15% of all breast cancers, differs from other subtypes [18].

Most comparative studies of breast cancer subtypes consider clinical or biological variables for classifying samples. However, these studies fail to account for the heterogeneity of samples within subtypes as well as clonal origin of most tumors. For example, although cultured TNBC cell lines routinely used in the laboratory are similar in the context of receptor status, they are distinct in terms of their genotypes. Human breast tumors similarly show significant genetic heterogeneity. Thus, studies involving samples from TNBC and non-TNBC cancer subtypes with diverse genetic background [19] can’t be directly compared, especially when most breast cancers start as clonal in the initial stage of tumor formation. To mitigate this issue and to eliminate variability due to different genetic backgrounds, we established an isogenic cell line model system representing two common breast cancer subtypes. We stably transfected empty vector or HER2 in two TNBC cell lines MDA-MB-231 and MDA-MB-468 and created isogenic TNBC or non-TNBC differing in the status of HER2. After initial characterization of these isogenic cell lines, we performed a microarray-based gene expression profiling to deduce the signatures of TNBC compared to non-TNBC isogenic cells, and searched resulting signatures in compactable publically available data sets.

## Methods

### Generation of Stable Clones

Triple-negative breast cancer MDA-MB-231 and MDA-MB-468 cells (ATCC) were chosen for stable clone generation. Origin of the cell lines have been described previously [19]. Transient transfections with 2.5-10 µg of plasmid per reaction with Fugene transfection reagent (Roche Ltd.) were used to optimize transfections. Using optimal conditions, the two cell lines were transfected with each of the two plasmids; pcDNA 3.1a and HER2. Cells were cultured using Dubelcco’s Modified Eagle Medium/ Ham’s F12 50:50 (DMEM/F-12) mix (Mediatech) supplemented with 10% FBS (Atlanta Biologics) and 1% Antibiotics (Gibco) and kept at an incubator maintained at 37°C and 5% CO2. The transfected cells were then treated with 0.5µg/ml of G418 over several weeks to select for the cells containing the plasmids. Multiple plates were then pooled and selected for 2-3 more weeks to generate multiple stable clones. Proteins from these plates were harvested using RIPA buffer and 50µg of protein were loaded on an 8% SDS-PAGE gel & transferred on a nitrocellulose paper (Biorad). TNBC and HER2+ clones generated on one type of cell line were included in one gel along with negative & positive controls. Protein from parental (untransfected) cell line SKBR3 (HER2-positive) cell lines were used as negative and positive controls respectively. The membrane was then blotted with HER2 antibody (Bethyl) and was reprobed with vinculin antibody (Sigma) as a protein loading control. Protein in the membrane was then detected using ECL reagent (GE Healthcare) and exposed onto an autoradiography film (Hyblot CL). Clones showing highest expression of HER2 protein compared to negative control were selected for further experiments. Our experimental studies involved established *in vitro* immortalized human breast cancer cell lines and secondary data from in-house RNA-sequencing study and public microarray repository of human patient samples. Therefore, an ethical approval was not needed.

### Flow Cytometry

Cells were plated in duplicates in 60 cm dishes with complete media and allowed to grow at 37°C and 5% CO2 until the cells reached desired confluency. After one wash with PBS, the cells were treated with 0.5M EDTA to detach cells, followed by centrifugation at 500 x g for 5 minutes. They were then washed thrice with PBS buffer containing 0.5% BSA and resuspended in the same buffer to get approximately 4X10^^6^ cells/ml. From this, about 10^^5^ cells in a reaction volume of 25µl were taken and added to a tube containing 10µl of Phycoerythrin (PE) conjugated anti-human HER2 antibody (R&D Systems). For isotype control, 10 µl of PE-conjugated mouse IgG2B reagent (R&D Systems) was added to 10^^6^ cells. The mixture was incubated for about 45 minutes at 4°C. The cells were washed twice with PBS buffer containing 0.5% BSA and resuspended in 200-400µl of PBS for flow cytometry analysis. For experiments involving Herceptin treatment, two HER2 and a TNBC clone were plated in 60 cm dishes. Starting the next day the cells were serum starved for 24 hours. After starvation, cells were treated with 10nM Herceptin and incubated at 37°C and 5% CO2 for 16 hours. Cells were then collected and prepared for flow cytometric analysis as mentioned above.

### Confocal Microscopy

Cells were plated in 60 cm dishes with complete media, and, starting the next day, serum starved for 24 hours. After starvation, cells were treated with 10nM Herceptin and incubated at 37°C and 5% CO_2_ for 16 more hours. After one wash with PBS, the cells were trypsinized and plated over glass cover slips placed on culture plates. The cells were then fixed in 4% paraformaldehyde for 20 minutes at room temperature, permeabilized for 5-15 min with 0.1% triton-X-100. Indirect immunofluorescence technique was used to examine the cells. The cells were blocked with 5% normal goat serum for half an hour and then incubated with HER2 antibody (1:50 dilution) for 2 hours at room temperature, washed three times with PBS, and incubated with Alexa Fluor 546-labeled secondary antibody (Molecular Probes). We used DAPI (Molecular Probes) to stain DNA. Confocal microscopy was performed using a Zeiss laser-scanning confocal microscope.

### Gene Expression Profiling Using Microarray

Triplicates of one each of TNBC and HER2+ isogenic clones in both cell lines were plated and grown to 60-70% confluency in complete media containing G418. RNA was extracted using TRIZOL reagent (Invitrogen) according to manufacturer recommendations and quantified using a Nanodrop. Using RNeasy Mini Kit (Qiagen, Valencia, CA), RNA was purified and its integrity was tested using Agilent 2100 Bioanalyzer (Agilent Technologies, Santa Clara, CA). After RNA cleanup and labeling, the samples were hybridized onto an Affymetrix Human Exon 1.0 ST array chip and washed according to manufacturer’s protocol. The chips were then scanned to measure signal intensities. Resulting raw files were preprocessed using Robust Multi-array Average (RMA) algorithm, filtered and normalized by quantile technique using Gene Spring GX 10.0 (Agilent Technologies Inc.). Unpaired t-test was used to identify statistically significant differentially expressed genes between TNBC and HER2+ (p-value ≤0.05 and fold-change ≥1.5) in each cell line background. Benjamini and Hochberg method was used for multiple testing correction.

### Validation by quantitative Real Time PCR

RNA was extracted from the cells using TRIZOL reagent (Invitrogen) and 1µg was used for cDNA synthesis using Superscript III reverse transcriptase kit (Invitrogen) with oligodT method. Using SYBR Green RT PCR mastermix (Biorad), the qPCR reaction was set up in duplicates using 1µl of the cDNA as a template. 18S was used as a housekeeping control. The fluorescence detection and measurements were taken using Applied Biosystems thermal cycler. The relative expression levels of candidate genes for each cell line were calculated after normalization with control. The resulting values were then averaged and plotted as bar plot. Standard error (S.E.) was included in the graph. Two tailed unpaired student’s t-test was used for statistical analysis of the difference in expression between TNBC and HER2 clones.

### Comparison with Microarray from GEO

Among the breast cancer microarray datasets with patient samples in Gene Expression Omnibus (GEO) [20], studies employing GPL96 (Affymetrix Human Genome U133A Array) and GPL570 platforms (Affymetrix Human Genome U133 Plus 2.0 Array) were searched. Datasets containing samples from patients with untreated tumors or tumors prior to treatment were chosen. Samples representing the subtypes included in our study were selected based on the clinical annotation and information provided by Lehmann et al in identifying tumor subtypes of the samples [21]. Two different datasets representing each of the two microarray platforms (GPL96 and GPL570) were compiled for comparison with our results. Datasets we included in our study were GSE7390, GSE2603, GSE3494, GSE2990, GSE2034, GSE11121, GSE1561 and GSE20194 from GPL96 platform. Similarly datasets from GPL570 platform were GSE7904, GSE2109, GSE19615 and GSE12276. Tables S1A and B provide information about the GEO datasets that were selected and number of samples for each subtype included from each dataset.

### Gene Ontology and Pathway Analysis

The gene ontology (GO) and pathway analysis of the genes with deregulated expression and splicing was analyzed using Database for Annotation, Visualization and Integrated Discovery (DAVID) [22,23]. The enrichment of GO terms comprising molecular process and biological functions were identified. A p-value of 0.05 was considered significant for the results.

## Results

### Establishing Isogenic Stable Cell line Models

**Figure 1:**
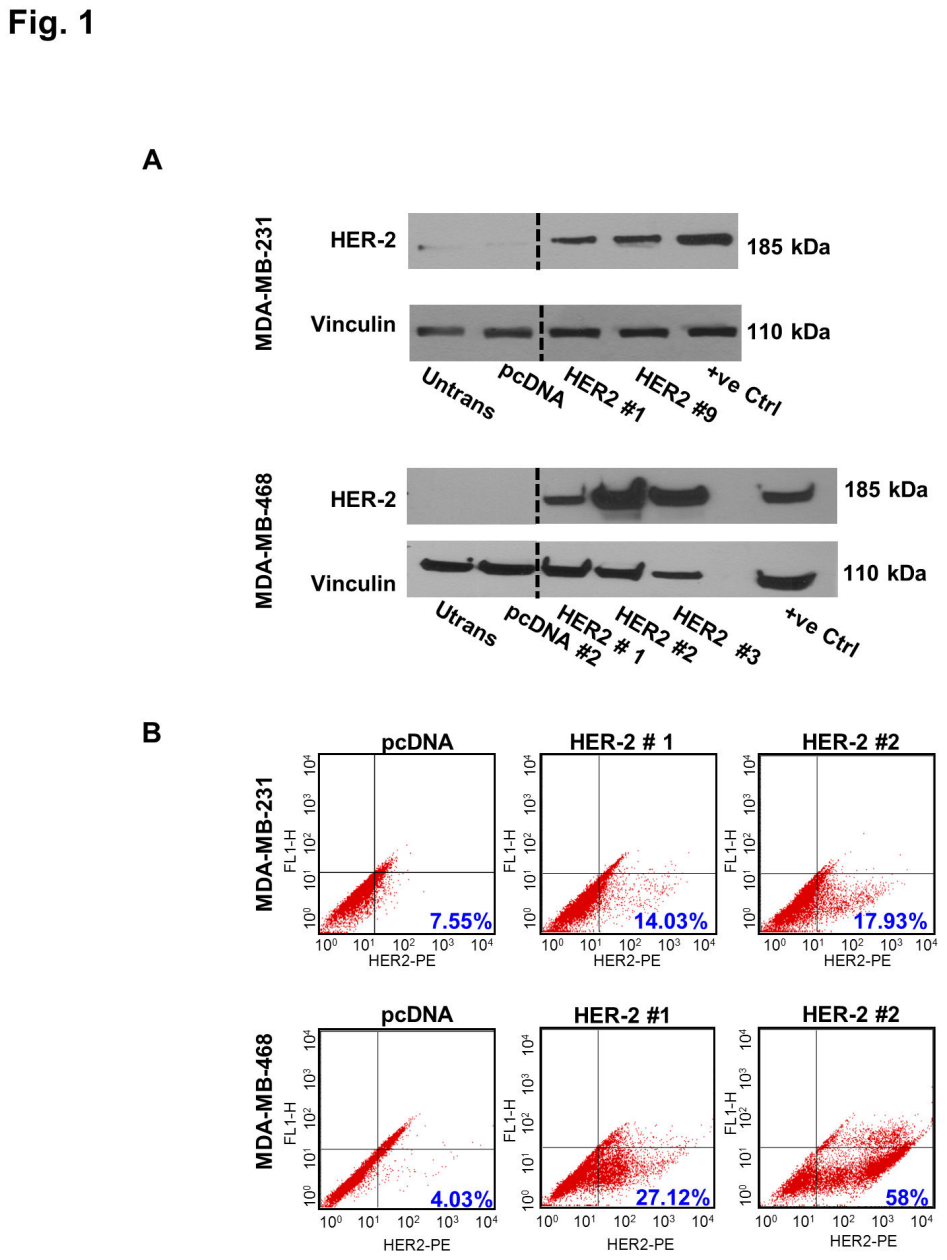
Expression of HER2 in stable isogenic clones. Expression level of HER2 in the receptor positive clones (HER2#1 and 9 in MDA-MB-231 and HER2#1, 2 and 3 in MDA-MB-468) was higher than in TNBC clones (pcDNA) in both cell line backgrounds. A) Representative western blot of stable cell lines showing HER2 expression. Two and three HER2 clones were selected for experiments based on initial assessment of receptor expression levels in HER2 clones compared to TNBC. 50µg of whole cell lysate was used for immunoblotting. Untransfected parental cell line (untrans.) was used as a negative control and HER2 overexpressing cell line SKBR3 was used as positive control (+ve control). Vinculin was used as a protein loading control. Black dotted lines indicate intervening lanes that have been removed. Total protein expression in HER2+ clones were higher than in TNBC clones and almost comparable to levels expressed in the positive control. B) Flow cytometry results of surface expression of HER2 in TNBC and HER2 positive clones. The percentage of cells with higher expression of HER2 is more in HER2 positive clones compared to TNBC clones. Numbers in blue indicate the percentage of the cells in the lower right quadrant. *Untrans*, untransfected; *+ve*, positive.

We created an isogenic model system for comparative study of two major breast cancer subtypes by stably transfecting empty vector or HER2 plasmids into TNBC cell lines MDA-MB-231 and MDA-MB-468. Protein expression levels of the reconstituted receptor in the pooled stable clones were measured using western blot. Results showed multiple clones in which the levels of HER2 in corresponding stable cell lines were higher than that in TNBC clones. Representative immunoblot of selected clones in each background showing high levels of HER2 in comparison to parental cell lines and TNBC clones are shown in Figure 1A. Surface expression of HER2 in TNBC and HER2+ clones was examined using flow cytometry. As shown in Figure 1B, HER2+ clones showed a larger population of cells with high expression of HER2 compared to TNBC clones.

### Biological Characterization of TNBC and HER2+ Isogenic Clones

**Figure 2:**
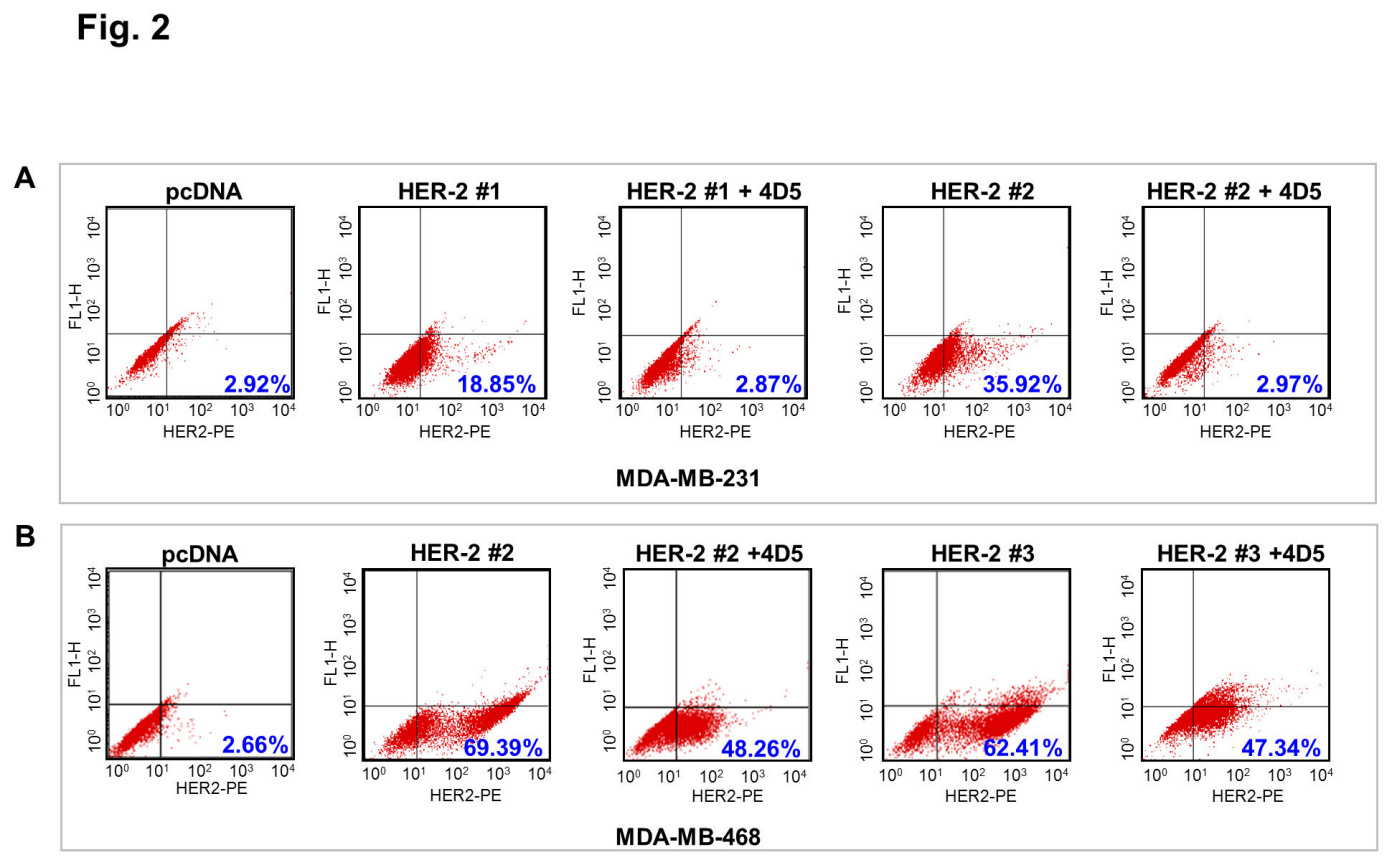
Downregulation of HER2 in TNBC and HER2+ve clones with Herceptin treatment. Flow cytometry was used to examine the levels of surface expression of HER2 in TNBC and HER2+ve clones in A) MDA-MB-468 and B) MDA-MB-231 backgrounds with or without Herceptin treatment. One TNBC and two HER2 clones in each cell line were treated with 10nM 4D5 after 24hr starvation. The percentage of cells with high expression of HER2 is decreased in HER2 positive clones after treatment with Herceptin. Numbers in blue indicate the percentage of cells in the lower right quadrant.*4D5*,Herceptin.

Next we examined whether the transfected HER2 receptor is responsive to anti-HER2 monoclonal antibody Herceptin. Treatment with Herceptin resulted in downregulation of surface expression of HER2 in the receptor positive clones in both cell lines as noted in the flow cytometry results (Figure 2). Numerical value in blue in the lower right of the flow cytometry measurements for each condition is the percentage of cells with high expression of HER2 as measured in that quadrant. The levels of the receptor tyrosine kinase after treatment of HER2+ clones with Herceptin were almost similar to the levels seen in TNBC clones. Congruent results were obtained using confocal microscopy as shown in Figure S1A-B.

### Gene Expression Analysis

**Figure 3:**
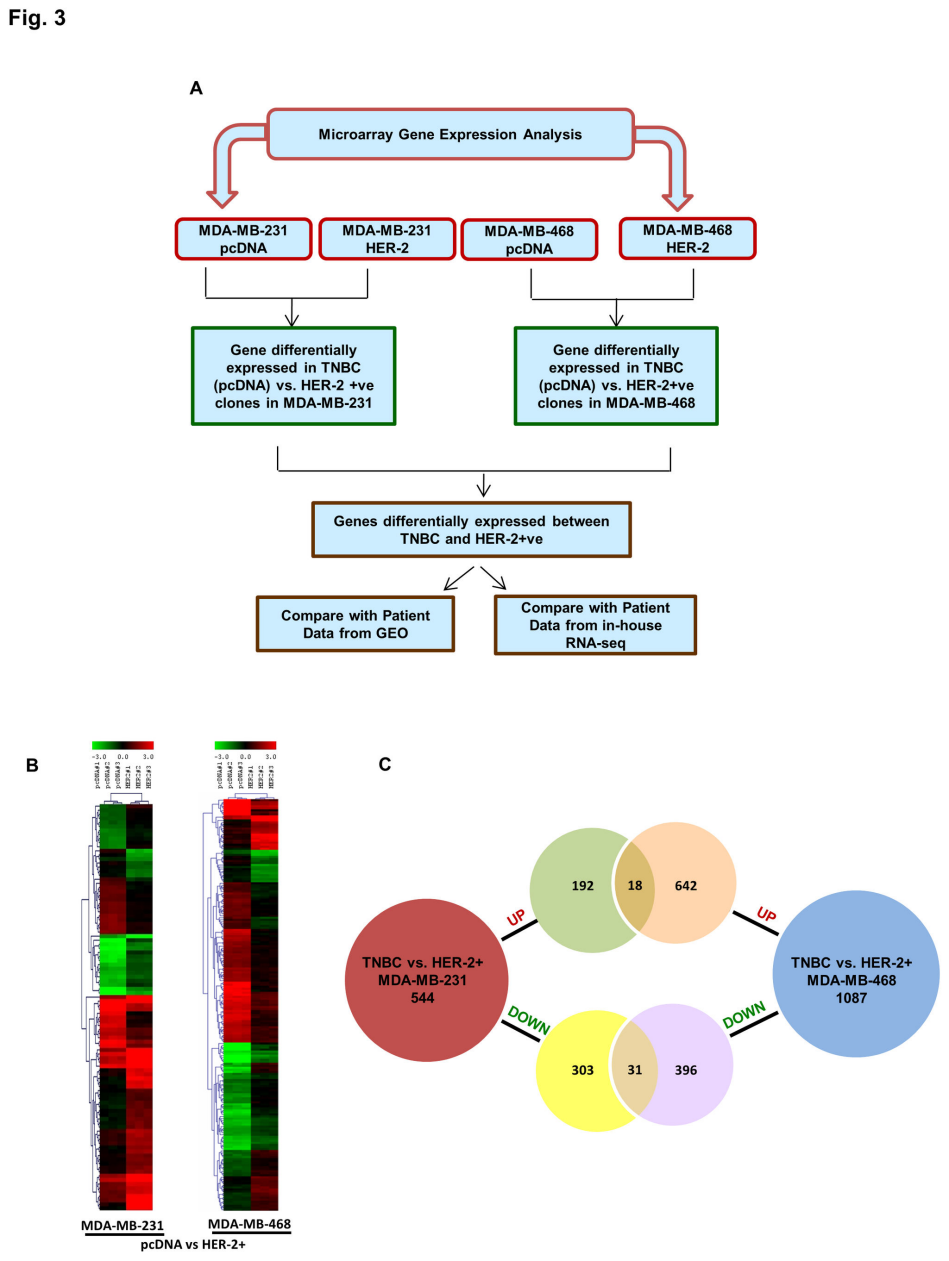
Gene expression profiling of stable clones using microarrays. A) A flow diagram for analysis of gene expression data from microarray experiment. Pairwise comparison of differential expression between TNBC and HER2+ clones are computed using a p value of 0.05 and Fold change 1.5. B) Heatmap showing clustering of differentially expressed genes between TNBC and HER2+ve samples. C) Venn diagram showing genes that are upregulated or downregulated in each cell line. Overlapping genes between the two cell lines are shown in the intersection of the Venn diagram.

Following biological characterization, we carried out gene expression profiling of TNBC and HER2+ clones in MDA-MB-231 and MDA-MB-468 background using an Affymetrix human exon array. Pairwise differential expression between the TNBC and HER2+ clones in both backgrounds were performed. A schematic diagram of differential expression analysis is illustrated in Figure 3A. In brief, pairwise differential expression analyses between the TNBC and HER2+ clones were performed individually for each cell line background and genes upregulated and downregulated in TNBC were identified. Gene lists from both backgrounds were then compared to delineate common genes with a similar expression pattern. Results from our studies were also overlaid with data from patient studies with similar breast cancer subtypes. A heatmap of the statistically significant differentially expressed genes (p. value ≤0.05 and fold change ≥1.5) in both backgrounds are shown in Figure 3B. Comparison of TNBC versus HER2+ clones in MDA-MB-231 cell lines resulted in 544 differentially expressed genes, 210 up and 332 downregulated. Similar comparison in the MDA-MB-468 cell lines also provided 1087 differentially expressed genes, 660 up and 426 downregulated (Figure 3C, Table S2). Between the two TNBC versus HER2+ comparisons, there were 49 genes that were common following same trend of regulation, with 18 upregulated and 31 downregulated genes (Figure 3C, Table 1).

**Table 1:** Deregulated genes in TNBC compared to HER2+ in MDA-MB-231 and MDA-MB-468 backgrounds.

Regulation	Gene	Description
Up	CASP2	caspase 2, apoptosis-related cysteine peptidase
Up	CCDC69	coiled-coil domain containing 69
Up	CDH1	cadherin 1, type 1, E-cadherin (epithelial)
Up	COBLL1	COBL-like 1
Up	CTSH	cathepsin H
Up	FNBP1L	formin binding protein 1-like
Up	KAL1	Kallmann syndrome 1 sequence
Up	KIAA1161	KIAA1161
Up	LRIG1	leucine-rich repeats and immunoglobulin-like domains 1
Up	PQLC3	PQ loop repeat containing 3
Up	PTGS1	prostaglandin-endoperoxide synthase 1 (prostaglandin G/H synthase and cyclooxygenase)
Up	SCARA3	scavenger receptor class A, member 3
Up	SELENBP1	selenium binding protein 1
Up	TMEM87B	transmembrane protein 87B
Up	TRIB2	tribbles homolog 2 (Drosophila)
Down	AGPAT5	1-acylglycerol-3-phosphate O-acyltransferase 5 (lysophosphatidic acid acyltransferase, epsilon)
Down	AGPAT9	1-acylglycerol-3-phosphate O-acyltransferase 9
Down	AHNAK2	AHNAK nucleoprotein 2
Down	ALDH1A3	aldehyde dehydrogenase 1 family, member A3
Down	CA9	carbonic anhydrase IX
Down	CTSB	cathepsin B
Down	DPYSL2	dihydropyrimidinase-like 2
Down	DUSP1	dual specificity phosphatase 1
Down	EDN1	endothelin 1
Down	EIF3E	eukaryotic translation initiation factor 3, subunit E
Down	ERRFI1	ERBB receptor feedback inhibitor 1
Down	EXOSC2	exosome component 2
Down	GDF15	growth differentiation factor 15
Down	GNA15	guanine nucleotide binding protein (G protein), alpha 15 (Gq class)
Down	GPRC5A	G protein-coupled receptor, family C, group 5, member A
Down	IRAK3	interleukin-1 receptor-associated kinase 3
Down	LOX	lysyl oxidase
Down	NOG	noggin
Down	NOV	nephroblastoma overexpressed
Down	NRK	Nik related kinase
Down	PAPPA	pregnancy-associated plasma protein A, pappalysin 1
Down	PRSS23	protease, serine, 23
Down	RGS2	regulator of G-protein signaling 2, 24kDa
Down	TMEM45B	transmembrane protein 45B
Down	TNFRSF10D	tumor necrosis factor receptor superfamily, member 10d, decoy with truncated death domain
Down	TNFRSF11B	tumor necrosis factor receptor superfamily, member 11b
Down	XK	X-linked Kx blood group (McLeod syndrome)

**Figure 4:**
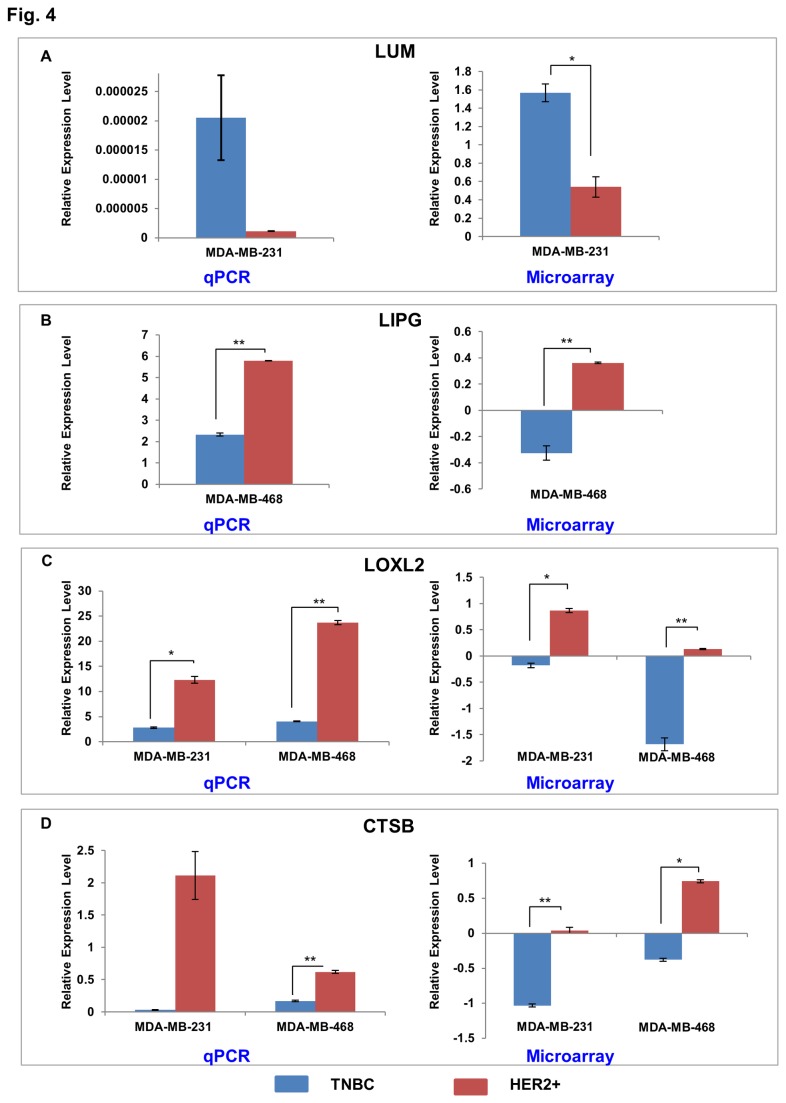
Validation of microarray results. Selected genes showing significant deregulation (p-value ≤0.05 and fold change ≥ 1.5) of mRNA expression between TNBC and HER2 clones from microarray data were validated using qPCR. Shown here are the expression levels of four candidates A) *LUM* B) *LIPG* C) *LOXL2* and D) *CTSB*. The expression levels measured by qPCR are shown in the left, while those from microarray are shown in the right. The expression values for qPCR were calculated using ΔΔ Ct method using 18S for normalization. Microarray values represent normalized and preprocessed data that have been log transformed. The plotted data represent mean ± S.E. Two-tailed student’s t-test was used for statistical analysis of qPCR data. Statistically significant differences in expression are indicated with *. Similar trend of regulation was observed for data from both techniques for these four genes. *, *p ≤0.05*; **, *p≤0*.*01*.

Based on biological significance and association with breast cancer, 34 candidates were selected from the differential expression gene lists for validation using qPCR. Among these 30 genes exhibited positive results showing a similar trend of expression levels as in the microarray analyses. Some of the candidates include Lumican (*LUM*), lipase, endothelial (*LIPG*), and Lysyl oxidase homolog 2 (*LOXL2*), Cathepsin B (*CTSB*) (Figure 4A-D). *LUM* is upregulated while LIPG is downregulated in the TNBC clones as compared to the HER2+ clones from MDA-MBA-231 and MDA-MB-468 cell lines, respectively. Moreover, *LOXL2* and *CTSB* are downregulated in the TNBC clones as compared to the HER2+ clones in both cell lines. The difference in expression levels were significant for *LIPG* (p <0.01), *LOXL2* in MDA-MB-231(p < 0.05) and MDA-MB-468 cell lines (p < 0.01), and for *CTSB* in MDA-MB-468 cells (p < 0.01).

*LUM* belongs to the small leucine rich proteoglycan family of proteins that is involved in collagen fibril organization, in growth and migration of epithelial cells, in tissue repair, and in the progression of breast tumor. Higher expression of LUM was found in breast cancer stroma as compared to the normal tissue and was found to be associated with low levels of estrogen receptor and higher tumor grade [24].

*LIPG* is a member of the triglycerides lipase family and is thought to be associated with metabolism of lipoproteins in endothelial cells. It was found to be one of the lipid metabolizing enzymes whose expression correlated with HER2 overexpression in a breast cancer cell line [25].

A member of the lysyl oxidase family, *LOXL2*, is of paramount importance in the extracellular matrix remodeling by crosslinking collagen with elastin. It is also found to play an important role in the development, tumor progression, epithelial to mesenchymal transition (EMT) and senescence. It is associated with distant metastasis and poor survival rates [26].

CTSB is a member of the lysosomal cysteine proteinases and is involved in protein degradation. It is found to play an important role in tumor invasion and metastasis and also considered a prognostic marker in an aggressive form of breast cancer known as the inflammatory breast cancer [27,28]. CTSB along with CTSL was found to be overexpressed in HER2 positive cancers and is an important mediator of tumor invasion in this subtype of breast cancer [29].

Next, we were interested in biological contextualization of the differentially expressed gene list that we obtained from microarray analysis. We did a gene ontology analysis using DAVID to investigate if any functional categories were enriched in our data. The top ten biological processes and molecular function of differentially expressed genes between the TNBC and HER2+ clones in each background are depicted in Table S3A-D, respectively. Biological functions like cell signaling, adhesion, regulation of apoptosis and proliferation were common themes for genes upregulated in TNBC in both cell lines. Similarly, shared themes like response to wounding and organic substance were common categories in genes downregulated in TNBC.

### Comparison with Data from Patient Samples

**Figure 5:**
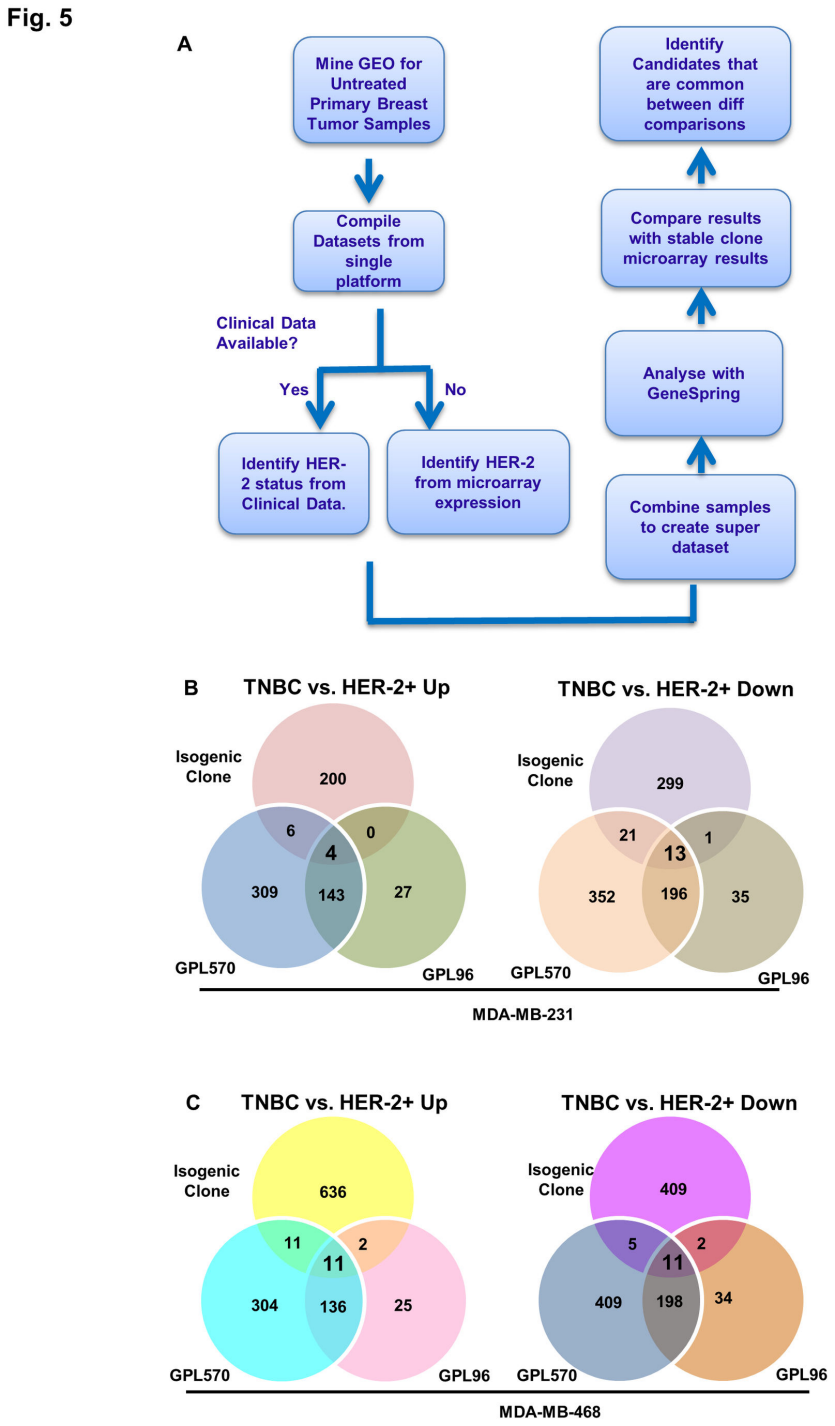
Comparison of microarray data from isogenic clones and patient samples. A) Flow diagram showing curation of microarray patient sample data from GEO repository and compilation of two super datasets. B) Results from pairwise differential expression comparison between TNBC and HER2+ve tumors in each of the super-datasets created from samples in GPL96 and GPL570 microarray platforms. C) Venn Diagrams showing up and downregulated genes in each of the two pairwise comparison between TNBC and HER2+ samples from isogenic clone data and the two GEO super-datasets. Various overlaps were seen in each comparison.

We next compared the differential gene expression signature derived from the isogenic studies with data obtained from patients with breast cancer of similar subtypes, i.e. TNBC vs. HER2+. We initially aligned our data to individual datasets from independent studies. However, due to variability in the overlaps between our results and the datasets being compared and small sample size in many of these datasets, we switched to a different approach. We sought to create a super-dataset from various microarray studies by different groups of investigators. We identified several studies from Gene Expression Omnibus (GEO) repository that contained microarray data from breast cancer patients that hadn’t undergone any treatment. During curation of datasets, we only included samples from studies that used two most common Affymetrix platforms in GEO (GPL96 and GPL570). Although the sample size of dataset containing samples from GPL96 platform was bigger, the probes included in the platform were almost half of those included in the GPL570 platform. Therefore, we created two independent super-datasets that included samples corresponding to our subtypes from the two platforms. Schematic diagram for curation of samples from GEO is shown in Figure 5A. Pairwise comparisons of deregulated genes between breast cancer subtypes from the two GEO super datasets are shown in Figure S2 (Table S4, S5).

Comparison of GEO datasets with data from MDA-MB-231 resulted in 4 up and 13 downregulated genes in TNBC vs. HER2+ (Figure 5B, Table S6). Analogously, 11 genes each were found to be up and downregulated in comparison with the MDA-MB-468 data (Figure 5C, Table S7). Overlap of these two comparisons provided two downregulated genes, GDF15 and GPRC5A; however, there were no upregulated genes.

**Figure 6:**
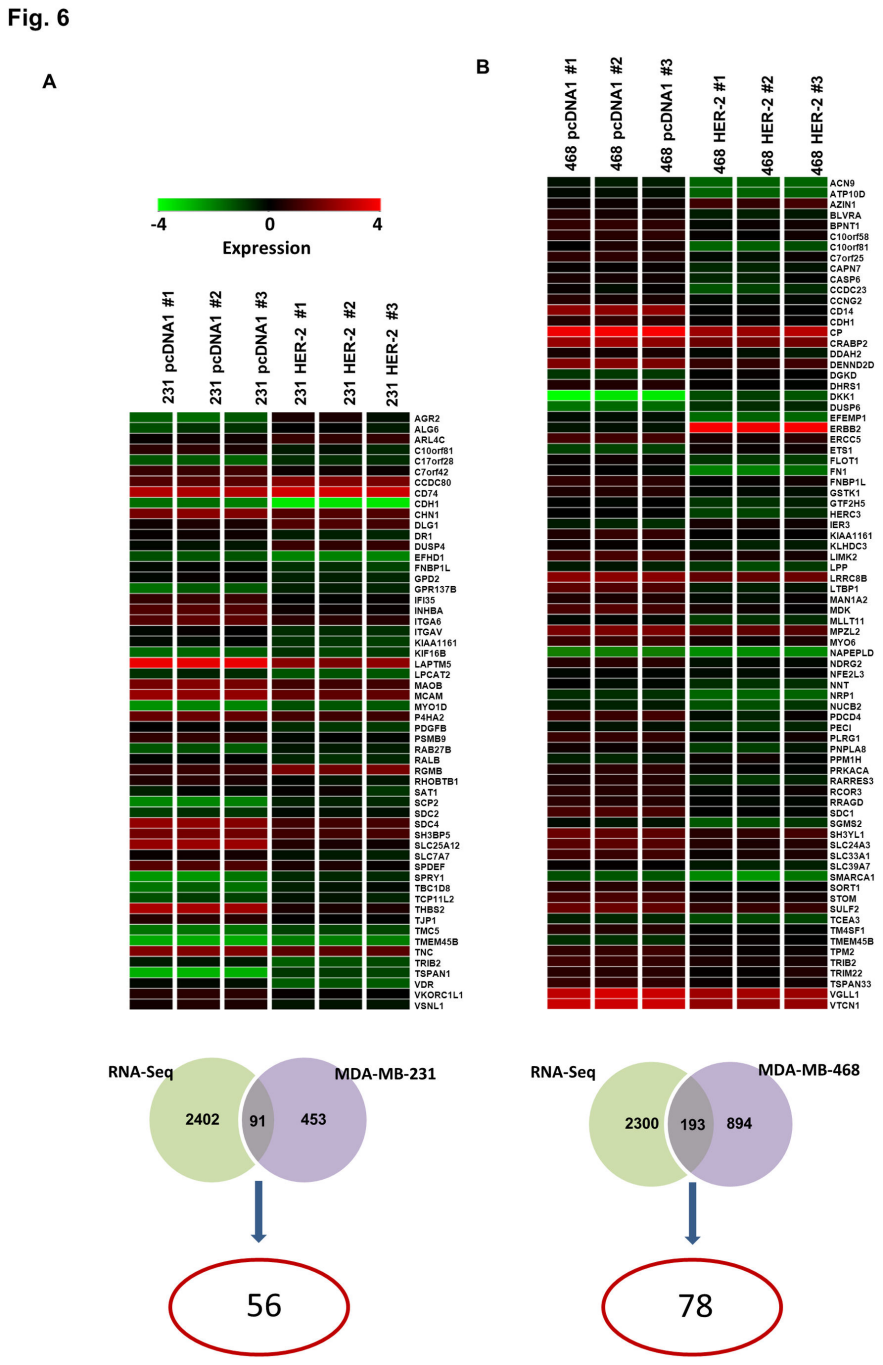
Comparison of microarray data from isogenic clones and RNA-seq data from patient samples with similar breast cancer subtypes. A) Comparison of differentially expressed genes obtained from RNA-seq data from patient samples and microarray data in A) MDA-MB-231 background B) MDA-MB-468 background. The overlap in the Venn diagram show common genes between the comparisons and the number circled in red indicates the candidates that show same trend of regulation in both microarray and RNA-seq dataset (shown as a heatmap).

Additionally, we also compared our results with a published mRNA sequencing based study of different breast cancer subtypes from our lab [30]. Comparison of MDA-MB-231 cells results with the sequencing data sets resulted in 56 genes that showed the same deregulation pattern in TNBC as compared to HER2+ samples (Figure 6A, Table S8a, b). Parallel comparison with data from MDA-MB-468 cells resulted in 72 genes with same trend of regulation (Figure 6B, Table S8 c, d). From these two comparisons, we found 10 common genes that followed the same regulation pattern.

## Discussion

We have established an isogenic model system for the comparative study of TNBC and non-TNBC (HER2+) subtypes. The reengineered non-TNBC cell lines express HER2 receptors at levels comparable to receptor-positive cell lines. As a proof of functionality of transfected receptor, we observed an effective downregulation of overexpressed HER2 in the stable clones after treatment with Herceptin. Furthermore, isogenic background of the stable cell lines made comparison between different subtypes feasible without any generally noticed variability of genetic background. To the best of our knowledge, this is the first isogenic cell line model for comparing two major breast cancer subtypes. Using our model system, we have identified gene expression signatures that differentiate TNBC and HER2+ breast cancer subtypes.

Our goal was to identify a genomic signature associated with the status of HER2 in breast cancer and how their loss in TNBC affects the expression of other genes. Using microarray technology, we interrogated the expression levels of multiple genes that changed as a result of expression of HER2 alone in isogenic setting using two different cell lines. We have characterized a signature of TNBC in comparison to HER2+, non-TNBC subtype. In addition, we have also identified a comprehensive list of all statistically significant deregulated genes between TNBC and HER2+ cell line in isogenic background. A survey of literature pointed out several candidates from our studies to be in line with various published studies, validating the merit of our study. We found that Fibroblast Growth Factor Receptor 2 (FGFR2) and acyl-CoA dehydrogenase, short/branched chain (ACADSB), which was found to be upregulated in TNBC in a study by Turner et al. to have higher expression in TNBC clones compared to two other subtypes [31]. Similarly Cysteine-rich angiogenic inducer 61 (CYR61), that we found to be upregulated in TNBC compared to HER2+ subtype, is overexpressed significantly in TNBC. CYR61 is also significantly upregulated in invasive breast cancer and considered as an important therapeutic target for breast cancer [32]. From the list of molecules that are positively correlated to HER2 status in breast cancer tumors and cell lines from a study by Bertucci et al., we found two candidates lysl oxidase (LOX) and fatty acid desaturase 2 (FADS2) with similar correlation in our data sets [33]. Candidates reported in this study could include molecules that are affected by the downregulation of HER2 in TNBC, including novel targets of HER2, and are important players in the development of TNBC and its invasive phenotype.

One theory as to how TNBC might evolve is that in early stages, breast tumor starts out as a hormone receptor positive benign lesion that depends on hormones (e.g. estrogen) for its growth and proliferation. During the course of its malignancy, the tumor develops hormonal independence, gradually loses the expression of estrogen receptor and becomes more aggressive. However, the mechanism of downregulation of the estrogen receptor is poorly studied due to lack of a suitable model system [34]. Clark et al. studied down regulation of estrogen receptor using wild type MCF-7 and its sublines that lose their receptor expression and hormone dependence [35]. Differentially expressed genes between TNBC and HER2+ samples in our model system potentially constitute the gene signature that changes as a result of tumor progression from being receptor, HER2 in this case, positive to receptor null. Similarly, since TNBC shows absence or low levels of HER2, our data could point to negative regulators of the receptor tyrosine kinase in TNBC. However, additional studies are needed to validate these tentative conclusions and to gain a mechanistic insight into the downregulation of the receptor. We found several molecules with repressor functions like ERBB receptor feedback inhibitor 1 (*ERRFI1*), grainyhead-like 1 ( *Drosophila* ) (*GRHL1*) and E3 ubiquitin-protein ligase RING2 (*RNF2*) upregulated in TNBC clones compared to other two subtypes. It would be interesting to study if any of these are responsible for the downregulation of HER2 receptors in TNBC.

The overlap of gene expression signature from our cellular model with microarray data sets from breast cancer patient samples of corresponding subtypes increases the confidence of our finding. Additionally it also provides an essential receptor status related gene dataset with physiological significance for further studies. This could be of high value as diagnostic and therapeutic targets for TNBC.

## Conclusion

Using an isogenic model system, we have shown that the underlying molecular differences between various breast cancer subtypes are evident at the level of gene expression. Our findings point to key molecules and events that are potentially linked to the biology of TNBC and explain how it differs from HER2+ subtype. The deregulated genes potentially represent the signature that changes as breast cancer progresses into a more aggressive TNBC phenotype. Our findings also exhibit how upregulation of a single gene could lead to whole range of molecular changes in the isogenic cells. Importantly, a portion of alterations in the expression levels in the model system also hold true in human TNBC and non-TNBC HER2, suggesting the presence of an element of isogenic signature within the generally noted heterogeneous expression patterns. We provide an important dataset and a model system for further exploration and testable hypothesis generation. Further in depth studies are needed for confirming our findings and using it for identifying cases of TNBC from non-TNBC that would aid in tailoring subtype specific therapies.

## Supporting Information

Figure S1 **Surface expression of transfected HER2 is effectively downregulated upon 4D5 (Herceptin) treatment.** Confocal microscopy was used to determine the surface expression of HER2 in isogenic clones in A) MDA-MB-231 B) MDA-MB-468 backgrounds. One TNBC and two HER2 clones in each cell line were treated with 10nM 4D5 after 24hr starvation. Immunofluorescence staining was used for examining HER2 expression (red) in the control and treated cells. HER2 was expressed at a higher level in HER2 clones in comparison to TNBC (pcDNA) clones. Treatment of the HER2 clones with 4D5 reduced the expression of HER2 similar to the levels seen in the TNBC clones. These observations were similar for clones in both cell lines.
(PPTX)

Figure S2 **Differential gene expression between pairwise comparison of TNBC and non-TNBC (ER+ and HER2+) samples from GEO datasets compiled from A) GPL96 and B) GPL570 microarray platforms.** Fold change of 1.5 and p value 0.05 were used as cut-offs during the comparison. Top boxes (purple) give the total number of deregulated genes in each comparison. Meanwhile, boxes in red and green give a breakdown of upregulated and downregulated genes respectively.
(PPTX)

Table S1 **Number of samples from each datasets belonging to TNBC and HER-2 positive subtypes included in our super-datasets from A) GPL96 B) GPL570 platforms.**
(DOCX)

Table S2 **Deregulated genes between pcDNA and HER2+ clones in MDA-MB-231 and MDA-MB-468 backgrounds.**
(XLSX)

Table S3 **Top ten biological processes enriched in genes deregulated in TNBC vs. HER2+ in MDA-MB-231 and MDA-MB-468 cell lines.**
(DOCX)

Table S4 **Deregulated genes in TNBC compared to HER2+ in GPL96 super-dataset.**
(XLSX)

Table S5 **Deregulated genes in TNBC compared to HER2+ in GPL570 super-dataset.**
(XLSX)

Table S6 **Overlap of deregulated genes in TNBC vs. HER2+ in MDA-MB231 isogenic clones and two GEO datasets (GPL96 and GPL570).**
(XLSX)

Table S7 **Overlap of deregulated genes in MDA-MB-468 TNBC vs. HER2+ in isogenic clones and two GEO datasets (GPL96 and GPL570).**
(XLSX)

Table S8 **Overlap between deregulated genes in TNBC vs. HER2+ in isogenic clones in MDA-MB-231 and MDA-MB-468 cell lines and Patient RNA-seq data.**
(XLSX)

## References

[1] Jemal A, Bray F, Center MM, Ferlay J, Ward E et al. (2011) Global cancer statistics. CA Cancer J Clin 61: 69-90. doi:10.3322/caac.20107. PubMed: 21296855. 21296855 10.3322/caac.20107

[2] DeSantis C, Siegel R, Bandi P, Jemal A (2011) Breast cancer statistics, 2011. CA Cancer J Clin 61: 409-418. PubMed: 21969133. 21969133 10.3322/caac.20134

[3] Society AC (2011) Breast Cancer: Facts & Figures 2011-2012. Atlanta: American Cancer Society, Inc.

[4] Perou CM, Sørlie T, Eisen MB, van de Rijn M, Jeffrey SS et al. (2000) Molecular portraits of human breast tumours. Nature 406: 747-752. doi:10.1038/35021093. PubMed: 10963602. 10963602 10.1038/35021093

[5] Sørlie T, Perou CM, Tibshirani R, Aas T, Geisler S et al. (2001) Gene expression patterns of breast carcinomas distinguish tumor subclasses with clinical implications. Proc Natl Acad Sci U S A 98: 10869-10874. doi:10.1073/pnas.191367098. PubMed: 11553815. 11553815 10.1073/pnas.191367098 PMC58566

[6] Sotiriou C, Neo SY, McShane LM, Korn EL, Long PM et al. (2003) Breast cancer classification and prognosis based on gene expression profiles from a population-based study. Proc Natl Acad Sci U S A 100: 10393-10398. doi:10.1073/pnas.1732912100. PubMed: 12917485. 12917485 10.1073/pnas.1732912100 PMC193572

[7] Sorlie T, Tibshirani R, Parker J, Hastie T, Marron JS et al. (2003) Repeated observation of breast tumor subtypes in independent gene expression data sets. Proc Natl Acad Sci U S A 100: 8418-8423. doi:10.1073/pnas.0932692100. PubMed: 12829800. 12829800 10.1073/pnas.0932692100 PMC166244

[8] Hu Z, Fan C, Oh DS, Marron JS, He X et al. (2006) The molecular portraits of breast tumors are conserved across microarray platforms. BMC Genomics 7: 96. doi:10.1186/1471-2164-7-96. PubMed: 16643655. 16643655 10.1186/1471-2164-7-96 PMC1468408

[9] Hedenfalk I, Duggan D, Chen Y, Radmacher M, Bittner M et al. (2001) Gene-expression profiles in hereditary breast cancer. N Engl J Med 344: 539-548. doi:10.1056/NEJM200102223440801. PubMed: 11207349. 11207349 10.1056/NEJM200102223440801

[10] Vincent-Salomon A, Gruel N, Lucchesi C, MacGrogan G, Dendale R et al. (2007) Identification of typical medullary breast carcinoma as a genomic sub-group of basal-like carcinomas, a heterogeneous new molecular entity. Breast Cancer Res 9: R24. doi:10.1186/bcr1822. PubMed: 17417968. 17417968 10.1186/bcr1666 PMC1868916

[11] Farmer P, Bonnefoi H, Becette V, Tubiana-Hulin M, Fumoleau P et al. (2005) Identification of molecular apocrine breast tumours by microarray analysis. Oncogene 24: 4660-4671. doi:10.1038/sj.onc.1208561. PubMed: 15897907. 15897907 10.1038/sj.onc.1208561

[12] van de Vijver MJ, He YD, van’t Veer LJ, Dai H, Hart AA et al. (2002) A gene-expression signature as a predictor of survival in breast cancer. N Engl J Med 347: 1999-2009. doi:10.1056/NEJMoa021967. PubMed: 12490681. 12490681 10.1056/NEJMoa021967

[13] Parker JS, Mullins M, Cheang MC, Leung S, Voduc D et al. (2009) Supervised risk predictor of breast cancer based on intrinsic subtypes. J Clin Oncol 27: 1160-1167. doi:10.1200/JCO.2008.18.1370. PubMed: 19204204. 19204204 10.1200/JCO.2008.18.1370 PMC2667820

[14] Guedj M, Marisa L, de Reynies A, Orsetti B, Schiappa R et al. (2012) A refined molecular taxonomy of breast cancer. Oncogene 31: 1196-1206. doi:10.1038/onc.2011.301. PubMed: 21785460. 21785460 10.1038/onc.2011.301 PMC3307061

[15] Chen JQ, Russo J (2009) ERalpha-negative and triple negative breast cancer: molecular features and potential therapeutic approaches. Biochim Biophys Acta 1796: 162-175. PubMed: 19527773. 19527773 10.1016/j.bbcan.2009.06.003 PMC2937358

[16] Dent R, Trudeau M, Pritchard KI, Hanna WM, Kahn HK et al. (2007) Triple-negative breast cancer: clinical features and patterns of recurrence. Clin Cancer Res 13: 4429-4434. doi:10.1158/1078-0432.CCR-06-3045. PubMed: 17671126. 17671126 10.1158/1078-0432.CCR-06-3045

[17] Boyle P (2012) Triple-negative breast cancer: epidemiological considerations and recommendations. Ann Oncol 23 Suppl 6: 12-v 10.1093/annonc/mds187. PubMed: 23012306. 10.1093/annonc/mds187 23012306

[18] Irvin WJ Jr., Carey LA (2008) What is triple-negative breast cancer? Eur J Cancer 44: 2799-2805. doi:10.1016/j.ejca.2008.09.034. PubMed: 19008097. 19008097 10.1016/j.ejca.2008.09.034

[19] Neve RM, Chin K, Fridlyand J, Yeh J, Baehner FL et al. (2006) A collection of breast cancer cell lines for the study of functionally distinct cancer subtypes. Cancer Cell 10: 515-527. doi:10.1016/j.ccr.2006.10.008. PubMed: 17157791. 17157791 10.1016/j.ccr.2006.10.008 PMC2730521

[20] Barrett T, Edgar R (2006) Gene expression omnibus: microarray data storage, submission, retrieval, and analysis. Methods Enzymol 411: 352-369. doi:10.1016/S0076-6879(06)11019-8. PubMed: 16939800. 16939800 10.1016/S0076-6879(06)11019-8 PMC1619900

[21] Lehmann BD, Bauer JA, Chen X, Sanders ME, Chakravarthy AB et al. (2011) Identification of human triple-negative breast cancer subtypes and preclinical models for selection of targeted therapies. J Clin Invest 121: 2750-2767. doi:10.1172/JCI45014. PubMed: 21633166. 21633166 10.1172/JCI45014 PMC3127435

[22] Huang da W, Sherman BT, Lempicki RA (2009) Systematic and integrative analysis of large gene lists using DAVID bioinformatics resources. Nat Protoc 4: 44-57. PubMed: 19131956. 19131956 10.1038/nprot.2008.211

[23] Huang da W, Sherman BT, Lempicki RA (2009) Bioinformatics enrichment tools: paths toward the comprehensive functional analysis of large gene lists. Nucleic Acids Res 37: 1-13. doi:10.1093/nar/gkp505. PubMed: 19033363. 19033363 10.1093/nar/gkn923 PMC2615629

[24] Leygue E, Snell L, Dotzlaw H, Hole K, Hiller-Hitchcock T et al. (1998) Expression of lumican in human breast carcinoma. Cancer Res 58: 1348-1352. PubMed: 9537227. 9537227

[25] Cadenas C, Vosbeck S, Hein EM, Hellwig B, Langer A et al. (2012) Glycerophospholipid profile in oncogene-induced senescence. Biochim Biophys Acta 1821: 1256-1268. doi:10.1016/j.bbalip.2011.11.008. PubMed: 22178194. 22178194 10.1016/j.bbalip.2011.11.008

[26] Barker HE, Chang J, Cox TR, Lang G, Bird D et al. (2011) LOXL2-mediated matrix remodeling in metastasis and mammary gland involution. Cancer Res 71: 1561-1572. doi:10.1158/1538-7445.AM2011-1561. PubMed: 21233336. 21233336 10.1158/0008-5472.CAN-10-2868 PMC3842018

[27] Watson CJ, Kreuzaler PA (2009) The role of cathepsins in involution and breast cancer. J Mammary Gland Biol Neoplasia 14: 171-179. doi:10.1007/s10911-009-9126-8. PubMed: 19437107. 19437107 10.1007/s10911-009-9126-8

[28] Nouh MA, Mohamed MM, El-Shinawi M, Shaalan MA, Cavallo-Medved D et al. (2011) Cathepsin B: a potential prognostic marker for inflammatory breast cancer. J Transl Med 9: 1. doi:10.1186/1479-5876-9-S2-O1. PubMed: 21199580. 10.1186/1479-5876-9-1 PMC3022726 21199580

[29] Rafn B, Nielsen CF, Andersen SH, Szyniarowski P, Corcelle-Termeau E et al. (2012) ErbB2-driven breast cancer cell invasion depends on a complex signaling network activating myeloid zinc finger-1-dependent cathepsin B expression. Mol Cell 45: 764-776. doi:10.1016/j.molcel.2012.01.029. PubMed: 22464443. 22464443 10.1016/j.molcel.2012.01.029

[30] Eswaran J, Cyanam D, Mudvari P, Reddy SD, Pakala SB et al. (2012) Transcriptomic landscape of breast cancers through mRNA sequencing. Sci Rep 2: 264 PubMed: 22355776. 22355776 10.1038/srep00264 PMC3278922

[31] Turner N, Lambros MB, Horlings HM, Pearson A, Sharpe R et al. (2010) Integrative molecular profiling of triple negative breast cancers identifies amplicon drivers and potential therapeutic targets. Oncogene 29: 2013-2023. doi:10.1038/onc.2009.489. PubMed: 20101236. 20101236 10.1038/onc.2009.489 PMC2852518

[32] Espinoza I, Liu H, Busby R, Lupu R (2011) CCN1, a candidate target for zoledronic acid treatment in breast cancer. Mol Cancer Ther 10: 732-741. doi:10.1158/1535-7163.MCT-10-0836. PubMed: 21393426. 21393426 10.1158/1535-7163.MCT-10-0836 PMC3857108

[33] Bertucci F, Borie N, Ginestier C, Groulet A, Charafe-Jauffret E et al. (2004) Identification and validation of an ERBB2 gene expression signature in breast cancers. Oncogene 23: 2564-2575. doi:10.1038/sj.onc.1207361. PubMed: 14743203. 14743203 10.1038/sj.onc.1207361

[34] Leonessa F, Boulay V, Wright A, Thompson EW, Brünner N et al. (1992) The biology of breast tumor progression. Acquisition of hormone independence and resistance to cytotoxic drugs. Acta Oncol 31: 115-123. doi:10.3109/02841869209088890. PubMed: 1622625. 1622625 10.3109/02841869209088890

[35] Clarke R, Dickson RB, Brünner N (1990) The process of malignant progression in human breast cancer. Ann Oncol 1: 401-407. PubMed: 2083184. 2083184 10.1093/oxfordjournals.annonc.a057790

