# Supplementary material for: Genomic Insights into Triple-Negative and HER2-Positive Breast Cancers Using Isogenic Model Systems
Source: PLoS One. 2013 Sep 23;8(9):e74993. doi: 10.1371/journal.pone.0074993 (PMC3781103; doi:10.1371/journal.pone.0074993)
Supplement: Figure S1 — Surface expression of transfected HER2 is effectively downregulated upon 4D5 (Herceptin) treatment. Confocal microscopy was used to determine the surface expression of HER2 in isogenic clones in A) MDA-MB-231 B) MDA-MB-468 backgrounds. One TNBC and two HER2 clones in each cell line were treated with 10nM 4D5 after 24hr starvation. Immunofluorescence staining was used for examining HER2 expression (red) in the control and treated cells. HER2 was expressed at a higher level in HER2 clones in comparison to TNBC (pcDNA) clones. Treatment of the HER2 clones with 4D5 reduced the expression of HER2 similar to the levels seen in the TNBC clones. These observations were similar for clones in both cell lines. (PPTX) [file pone.0074993.s001.pptx]

## Slide 1
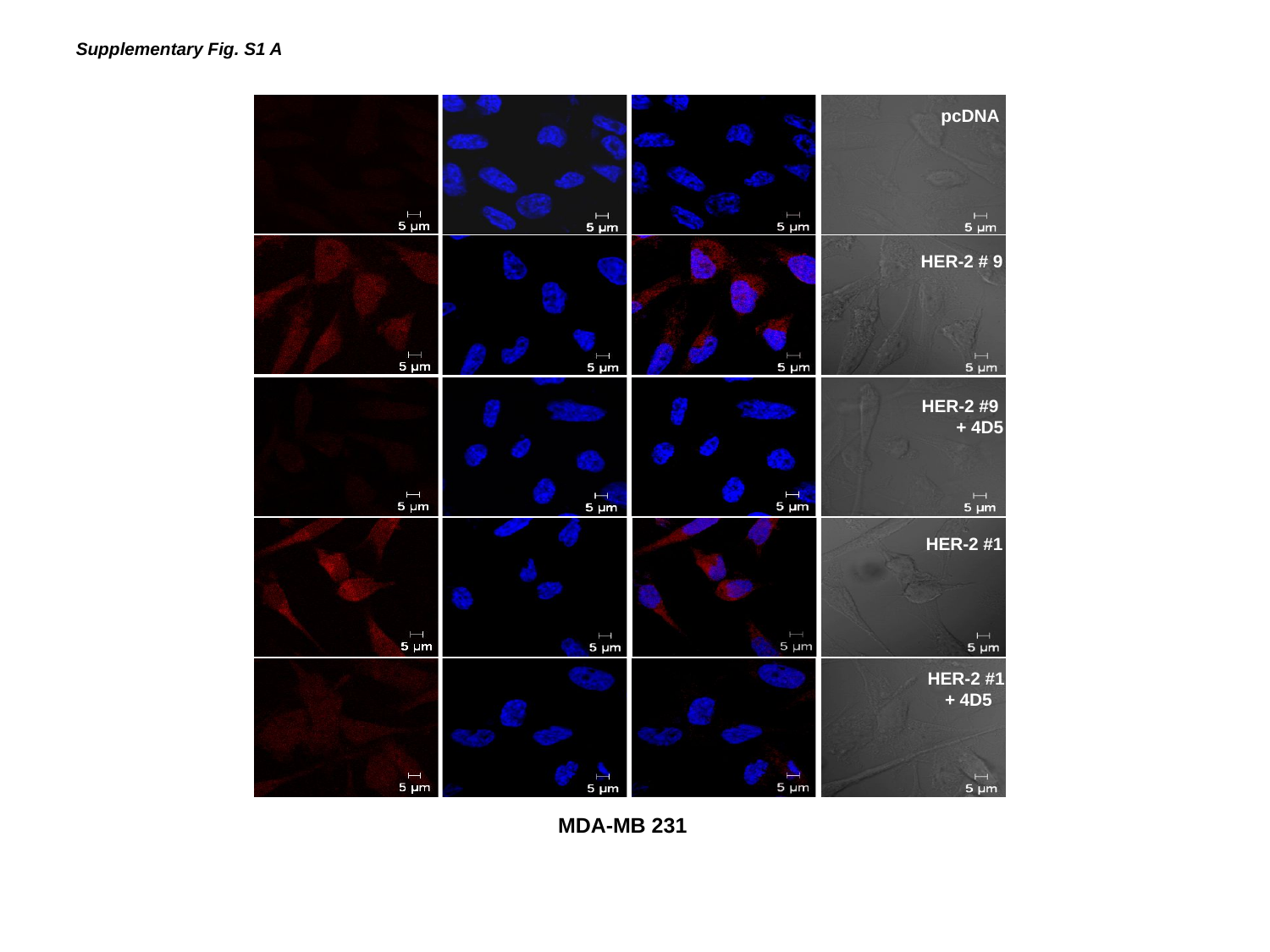

Supplementary Fig. S1 A
pcDNA
HER-2 # 9
HER-2 #9
+ 4D5
HER-2 #1
HER-2 #1
+ 4D5
MDA-MB 231

## Slide 2
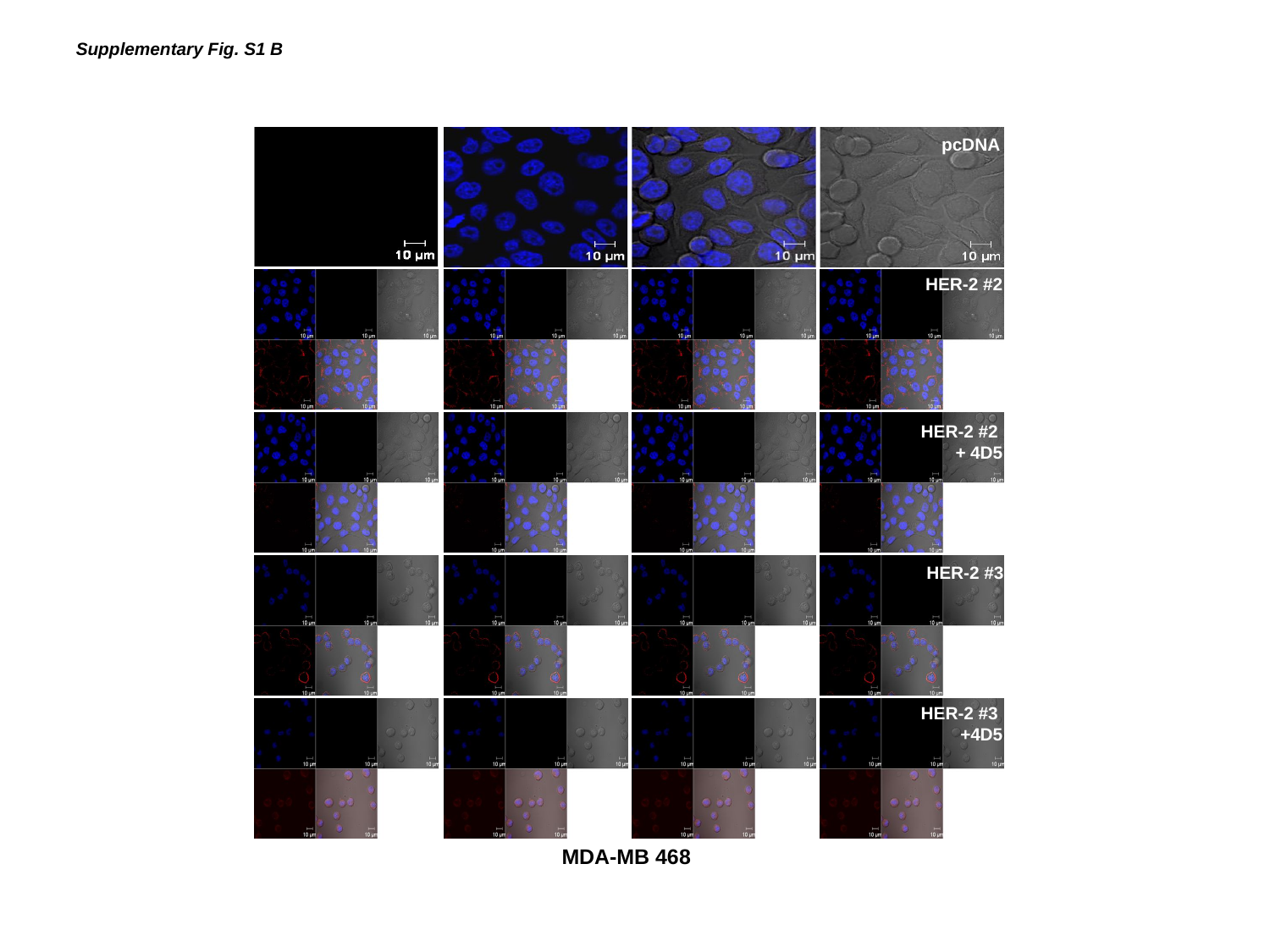

Supplementary Fig. S1 B
pcDNA
HER-2 #2
HER-2 #2
+ 4D5
HER-2 #3
HER-2 #3
+4D5
MDA-MB 468
