# Supplementary figures and images for: Genomic Insights into Triple-Negative and HER2-Positive Breast Cancers Using Isogenic Model Systems
Source: PLoS One. 2013 Sep 23;8(9):e74993. doi: 10.1371/journal.pone.0074993 (PMC3781103; doi:10.1371/journal.pone.0074993)

## Slide 1
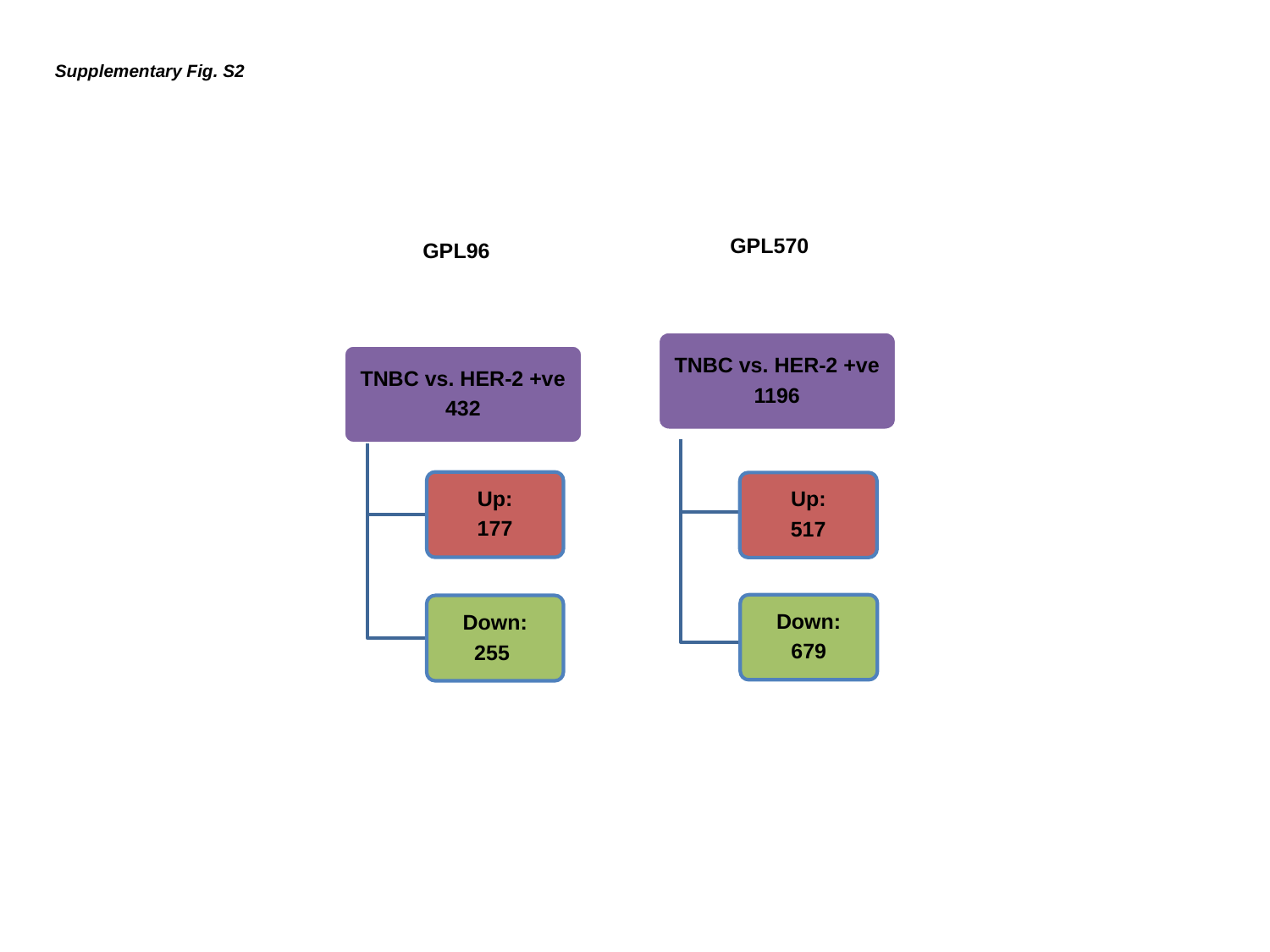

Supplementary Fig. S2
GPL570
GPL96
TNBC vs. HER-2 +ve
1196
TNBC vs. HER-2 +ve
432
Up:
177
Up:
517
Down:
679
Down:
255

Supplement: Figure S2 — Differential gene expression between pairwise comparison of TNBC and non-TNBC (ER+ and HER2+) samples from GEO datasets compiled from A) GPL96 and B) GPL570 microarray platforms. Fold change of 1.5 and p value 0.05 were used as cut-offs during the comparison. Top boxes (purple) give the total number of deregulated genes in each comparison. Meanwhile, boxes in red and green give a breakdown of upregulated and downregulated genes respectively. (PPTX) [file pone.0074993.s002.pptx]
