# Supplementary material for: Genomic Insights into Triple-Negative and HER2-Positive Breast Cancers Using Isogenic Model Systems
Source: PLoS One. 2013 Sep 23;8(9):e74993. doi: 10.1371/journal.pone.0074993 (PMC3781103; doi:10.1371/journal.pone.0074993)
Supplement: Table S1 — Number of samples from each datasets belonging to TNBC and HER-2 positive subtypes included in our super-datasets from A) GPL96 B) GPL570 platforms. (DOCX) [file pone.0074993.s003.docx]

**Table S1**

***Supplementary Table S1: Number of samples from each datasets belonging to TNBC and HER-2 positive subtypes included in our super-datasets from A) GPL96 B) GPL570 platforms.***

**A**

| **GPL96** | **No. of Samples** | |
| --- | --- | --- |
| **GEO ID** | **TNBC** | **HER-2 Positive** |
| GSE7390 | 39 | 12 |
| GSE2603 | 32 | 3 |
| GSE3494 | 30 | 21 |
| GSE2990 | 24 | 13 |
| GSE2034 | 56 | 19 |
| GSE11121 | 22 | 10 |
| GSE1561 | 18 | 4 |
| GSE20194 | 68 | 25 |
| ***Total*** | 289 | 107 |

| **GPL570** | **No. of Samples** | |
| --- | --- | --- |
| **GEO ID** | **TNBC** | **HER-2 Positive** |
| GSE7904 | 21 | 6 |
| GSE2109 | 94 | 28 |
| GSE19615 | 36 | 10 |
| GSE12276 | 64 | 22 |
| ***Total*** | 215 | 55 |

**B**
