# Supplementary material for: Genomic Insights into Triple-Negative and HER2-Positive Breast Cancers Using Isogenic Model Systems
Source: PLoS One. 2013 Sep 23;8(9):e74993. doi: 10.1371/journal.pone.0074993 (PMC3781103; doi:10.1371/journal.pone.0074993)
Supplement: Table S3 — Top ten biological processes enriched in genes deregulated in TNBC vs. HER2+ in MDA-MB-231 and MDA-MB-468 cell lines. (DOCX) [file pone.0074993.s005.docx]

**Table S3**

**Supplementary Table S3: Top ten biological processes enriched in genes deregulated in TNBC vs HER2+ in MDA-MB-231 and MDA-MB-468 cell lines.**


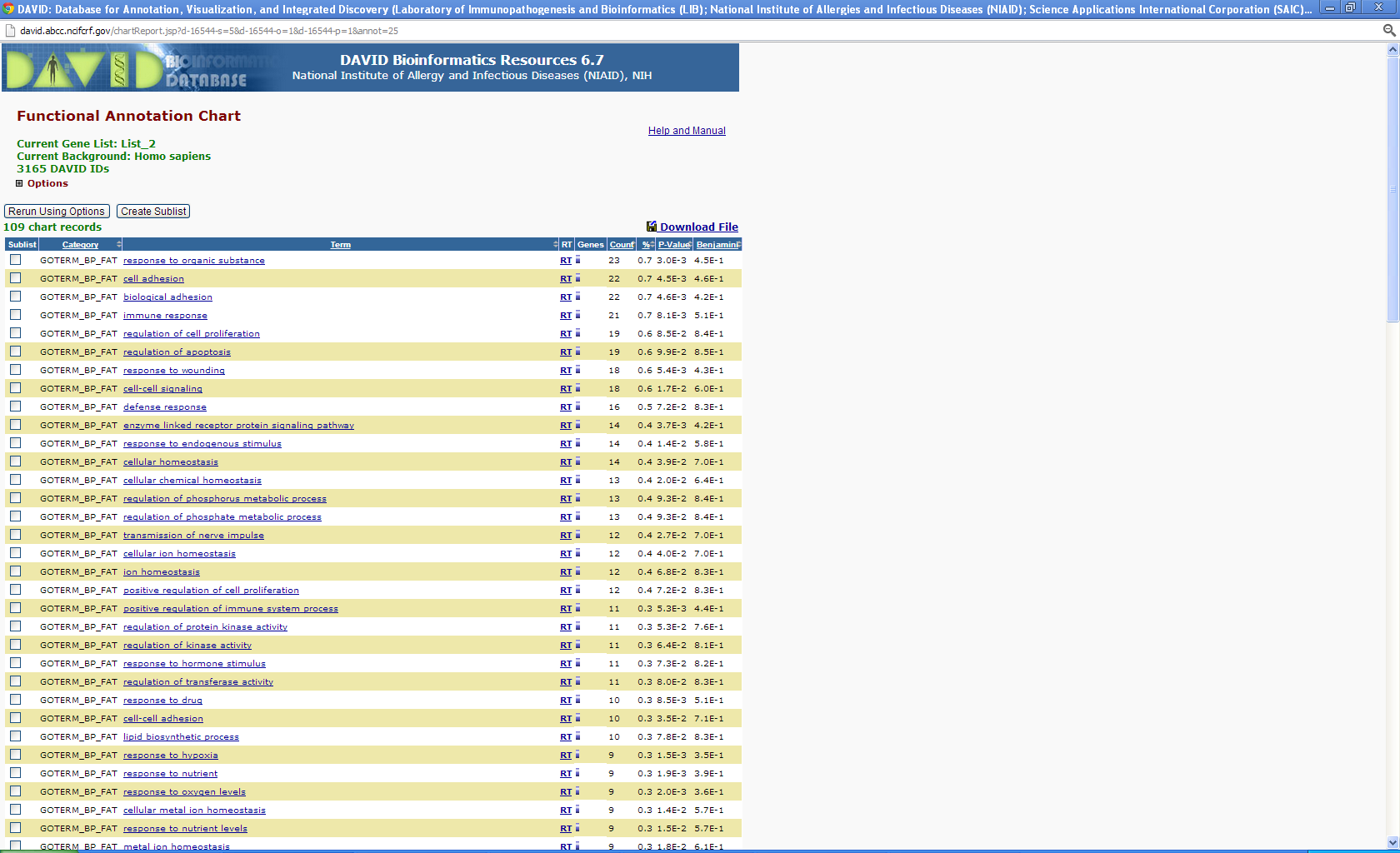


**B**

**MDA-MB-468 TNBC vs HER-2+ Up**

**A**

**MDA-MB- 231 TNBC vs HER-2+ Up**


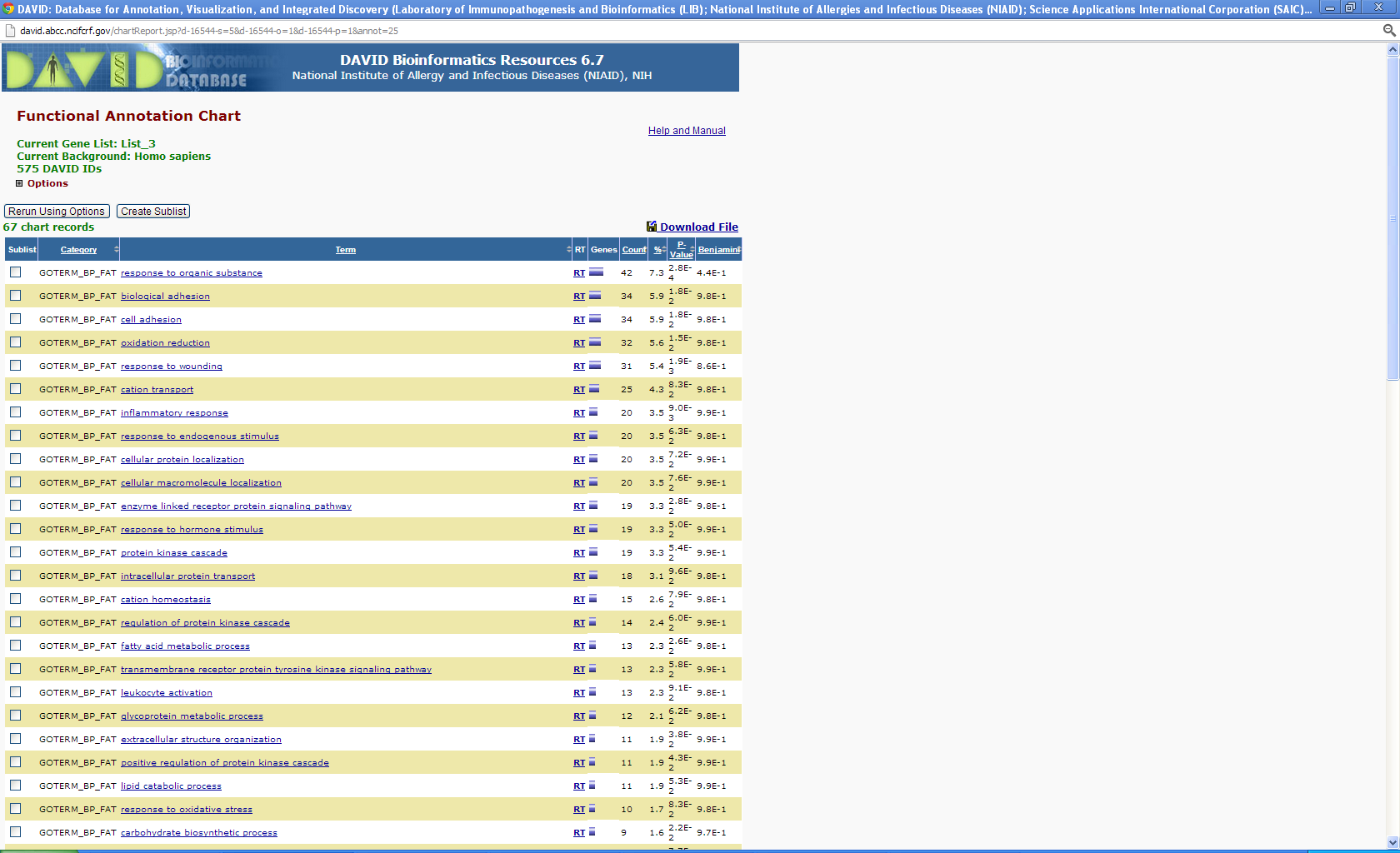

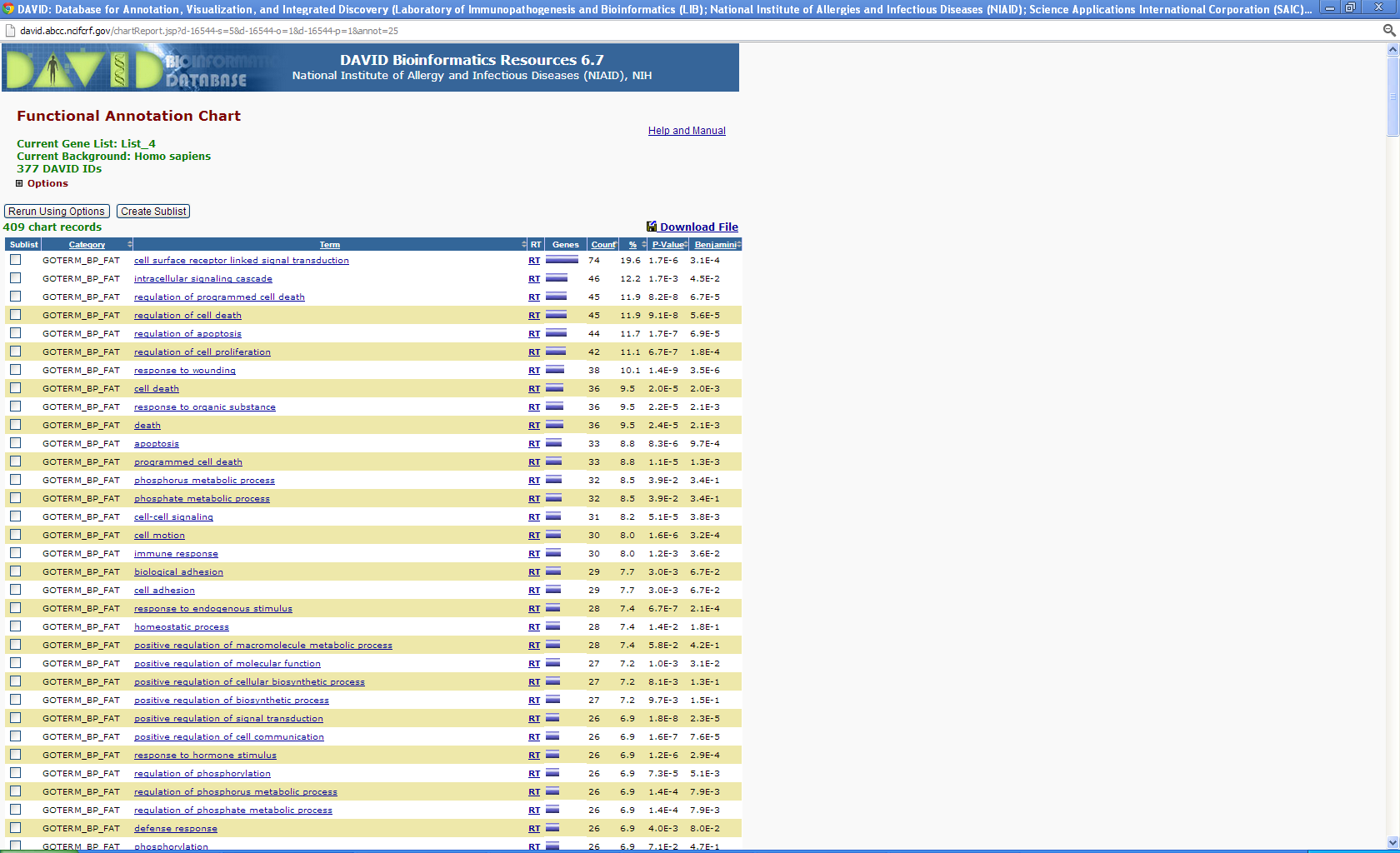


**C**

**MDA-MB-231 TNBC vs HER-2+ Down**

**D**

**MDA-MB-468 TNBC vs HER-2+ Down**
